# Public involvement and engagement in scientific research and higher education: the only way is ethics?

**DOI:** 10.1186/s40900-024-00587-x

**Published:** 2024-05-31

**Authors:** Claire Nollett, Matthias Eberl, Jim Fitzgibbon, Natalie Joseph-Williams, Sarah Hatch

**Affiliations:** 1 Centre for Trials Research, Cardiff University, 7th Floor, Neuadd Meirionnydd, Heath Park Campus, Cardiff, UK; 2 Division of Infection and Immunity, School of Medicine, Cardiff University, Cardiff, UK; 3 Systems Immunity Research Institute, Cardiff University, Cardiff, UK; 4 School of Medicine, Lead Public Contributor, Cardiff University, Cardiff, UK; 5 Division of Population Medicine, School of Medicine, Cardiff University, Cardiff, UK; 6 Health and Care Research Wales Evidence Centre, Cardiff, UK; 7 Public Involvement and Engagement Team, School of Medicine, Cardiff University, Cardiff, UK

**Keywords:** Public involvement, Public engagement, PPI, Ethics, Research ethics committee, Ethical approval

## Abstract

**Background:**

Involving and engaging the public in scientific research and higher education is slowly becoming the norm for academic institutions in the United Kingdom and elsewhere. Driven by a wide range of stakeholders including regulators, funders, research policymakers and charities public involvement and public engagement are increasingly seen as essential in delivering open and transparent activity that is relevant and positively impacts on our society. It is obvious that any activities involving and engaging members of the public should be conducted safely and ethically. However, it is not clear whether conducting activities ethically means they require ethical approval from a research ethics committee.

**Main body:**

Although there is some guidance available from government organisations (e.g. the UK Health Research Authority) to suggest ethical approval is not required for such activities, requests from funders and publishers to have ethical approval in place is commonplace in the authors’ experience. We explore this using case studies from our own institution.

**Conclusion:**

We conclude that any public-facing activity with the purpose to systemically investigate knowledge, attitudes and experiences of members of the public as research and as human participants requires prior approval from an ethics committee. In contrast, engaging and involving members of the public and drawing on lived experience to inform aspects of research and teaching does not. However, lack of clarity around this distinction often results in the academic community seeking ethical approval ‘just in case’, leading to wasted time and resources and erecting unnecessary barriers for public involvement and public engagement. Instead, ethical issues and risks should be appropriately considered and mitigated by the relevant staff within their professional roles, be it academic or a professional service. Often this can involve following published guidelines and conducting an activity risk assessment, or similar. Moving forward, it is critical that academic funders and publishers acknowledge the distinction and agree on an accepted approach to avoid further exacerbating the problem.

**Supplementary Information:**

The online version contains supplementary material available at 10.1186/s40900-024-00587-x.

## Abstract (plain language summary)

Involving and engaging members of the public is recognised best practice in university research and teaching. Involvement and engagement activities (for instance, working with the public to design a research study) continue to increase in priority and are an important part of an academic’s role. However, there is often confusion amongst researchers and educators around whether involving the public in these activities requires prior ethical approval, similar to what would be the case when inviting members of the public to participate in a clinical research study, or to donate samples such as blood for experiments. As an example, sometimes researchers are asked for ethical approval by scientific journals when trying to publish the findings from their public involvement and engagement work, when in fact this is not needed. The ongoing uncertainty about the difference between actual research on one hand and public involvement and engagement on the other hand wastes precious time and resources, and is a barrier for scientists to working with the public. We have developed guidance for academic staff on when ethical approval is and is not required, using examples from our own experience. We wrote this article to bring awareness to this problem; share our views with the wider academic community; encourage discussion around the problem and possible solutions; and ultimately contribute to educating on when research ethics approval is needed, and when not.

**Supplementary Information:**

The online version contains supplementary material available at 10.1186/s40900-024-00587-x.

## Background

Public involvement (PI) is ‘important, expected and possible in all types of health and social care research’ [1]. It is now commonly embedded and reported in health research papers in the UK, with approximately half mentioning public involvement activities [2]. Public engagement (PE) is also encouraged and recognised by funders and other stakeholders across the higher education sector to raise awareness, increase trust and transparency, share knowledge, foster learning and deliver positive impact to society [3].

In 2019, the UK Standards Partnership published the UK Standards for Public Involvement ‘to help researchers and organisations improve the quality and consistency of public involvement in health and care research’ [4], and a large knowledge base is developing around how to do public involvement well. However, PI is not without its challenges, as identified both in the literature e.g [5]. and through our own experience as academic researchers, professional services staff and members of several national public involvement committees. Key issues include how to efficiently pay and reimburse public contributors within organisations, how to effectively evaluate the impact, and how to provide inclusive opportunities and reach under-served groups to increase the diversity of those involved [6].

The Research Excellence Framework (REF) 2029, the UK’s national assessment of the quality of research produced by its higher education institutions held every 6–7 years, will see a 25% weighting of returns with respect to the social, economic and political influence of the research conducted. The 2029 round will in fact be the first REF assessment where impact will be measured as “*Engagement and* Impact” (our emphasis), alongside an accompanying statement to evidence engagement and impact activity beyond case studies [7]. As with PI, researchers face challenges in delivering PE including achieving the inclusion of under-served communities [8] and how to evaluate impact [3].

With individual researchers and their host institutions increasingly embracing PI and PE as part of their research and scholarship activities, there is one issue that we have found particularly contentious with researchers, employers, funders and publishers across both involvement and engagement and that is the focus of this commentary: the role of ethical approval in PI and PE activity.

Public involvement, sometimes referred to as Patient & Public Involvement (PPI) in health and social care research, is defined as ‘research being carried out ‘with’ or ‘by’ members of the public, rather than ‘to’, ‘about’ or ‘for’ them’ [9]. PE, adopting the UK’s National Coordinating Centre for Public Engagement’s definition, is a ‘myriad of ways in which the activity and benefits of higher education and research can be shared with the public’ [10]. PE is by definition a two-way process, involving interaction and listening, with the goal of generating mutual benefit. Both PI and PE are distinct from human participation in research whereby a member of the public agrees via informed consent to be a participant in research, e.g. receiving a study intervention, donating samples or sharing lived experiences. Whilst health and social care research involving human participants requires approval from a research ethics committee (REC), PI and PE activities typically do not.

In the UK, ethical approval is granted by a REC under the auspices of the National Health Service (NHS) for research on patients or healthcare professionals, or a local review committee or panel for research that does not include NHS patients. In academic research, this would usually be a university or school REC (referred to here as an Institutional Review Board, IRB). Other countries may use different approaches but the general need for RECs to approve research with human participants is ubiquitous. With regard to public involvement, the UK Health Research Authority (HRA) that is responsible for all NHS RECs explicitly states that ‘You do not need to submit an application to a Research Ethics Committee in order to involve the public in the planning or the design stage of research, even if the people involved are patients’ [11]. This advice would also apply to university ethics committees. However, despite this clear distinction, we have encountered and become aware of situations in which investigators were asked to acquire ethical approval for activities with the public – including PI, PE and impact activities. This highlights a potential misunderstanding of the nature of PI and PE, and their role alongside research. Whilst either activity can raise ethical considerations for the individuals involved, the requests to acquire *research* ethics approval for PI and PE need to be challenged within the academic community to increase awareness, understanding of and best practice around these activities. Seeking unnecessary approval adds a heavy additional burden on researchers which effectively acts as a barrier to carrying out PI and PE; can significantly delay timely activities; and uses valuable resources.

We propose that the requests to gain ethical approval for PI and PE activities stem largely from three main issues.

- Firstly, ‘grey’ areas, such as a blurring of the boundary between qualitative research and PI and PE activities, including confusion amongst the research community over the differences between research involvement, engagement and participation.
- Secondly, a perception amongst the research community that it is best to seek ethical approval ‘just in case’ or to ‘be on the safe side’, e.g. if asked by journal editors when trying to publish, rather than complete appropriate risk assessments to address any ethical considerations when carrying out PI and PE.
- And finally, lack of knowledge of an alternative recognised process on how to evidence that PI and PE activities with the public have been conducted in an ethical manner, if not approved by an NHS REC or local IRB.

Despite guidance indicating other ways to address ethical concerns in PI and PE [12–18], researchers, funders and publishers appear to be turning increasingly to university IRBs as the (perceived) ultimate arbiters of deciding ethical issues related to PI and PE activities. We see the need to highlight this as a growing problem and suggest ways the issues above can be overcome. We will firstly explore in more detail the distinction between qualitative research and PI and PE activities before outlining examples from our own experience around the three issues identified, and then proceeding to make recommendations for moving forwards.

## Public involvement and engagement vs. qualitative research

Distinguishing between whether activities with members of the public constitute PI and PE or qualitative research (and therefore require ethical approval) is a particularly ‘grey’ area [19]. This is especially true when consulting with a number of people at one time in what is usually referred to as a ‘focus group’. Going forward, it may be helpful to distinguish between ‘focus groups’, which are used for research, and ‘discussion groups’ used for PI and PE [20].

Several authors and organisations have described the difference between the two activities and developed useful side-by-side comparisons [19, 20]. In *focus groups* which are part of research, people attending are research participants who receive a standard Participant Information Sheet and provide informed consent. Their input will usually be recorded via an audio device, transcribed verbatim, treated as ‘data’, and systematically be analysed to answer a research question. For this, ethical approval is usually required. On the other hand, the contributions of people attending PI *discussion groups* will be recorded only as key points (e.g. a list of key themes emerging or key priorities discussed by the group in relation to a specific topic) to help shape and guide the research itself, such as agreeing which research outcome measures to use, helping to shape the intervention or the development of data collection materials like participant information sheets or interview guides. PI discussion groups do not require ethical approval but should be conducted in an ethical manner. Those involved should still be provided with information about the activity up front to ensure they are clear what their involvement will entail, and they may be asked to provide agreement or consent, but not in the formally documented way required for research. This is discussed in more detail in the recommendations.

Another grey area concerns whether direct quotes gathered from people in a discussion group can be used in a publication. Whilst ethical approval is not required for this, we do advise gaining documented agreement if you wish to do this, e.g. an email from the group member agreeing to quotes being used in a publication to illustrate the key points identified (not as data). In some cases, researchers will need to combine PI activities with a qualitative research approach and there may be confusion regarding which activities require approval. For example, an investigator may wish to interview new mothers as research participants to get their views on motherhood (research participation). This would require ethical approval. But prior to interviews, they may want to involve a separate group of new mothers in a discussion to help shape the topic guide for the interviews (PI). This would not need ethical approval [21].

## The extent of the problem - examples from our own experience

Through requesting examples from colleagues on their experiences, we uncovered many different situations within our own institution highlighting a difference of opinion on whether research ethics should be sought for PI and PE activity. We here outline three examples, giving the background to the project, the activity undertaken and the issues encountered.

### Example 1

*Writing a training program with charity service users and staff – request from charity and publication to seek ethical approval from the university IRB for the project*.

This project involved service users and charity staff in writing a mental health training curriculum for staff to identify depression in service users. Staff and service user input was sought through online meetings and email feedback. The attendees gave their opinions (based on their lived experience) on what should be included in the curriculum, and the key points were summarised to inform curriculum development. The information they gave was not treated as data to answer a research question and was not systematically analysed using qualitative methods. In this respect, HRA state that ’if you are collecting opinions rather than study data, your activity is likely an involvement activity’ [22].

Regardless of the above considerations, the project lead was asked by third sector organisations to seek university IRB approval, to ensure the service users would be treated in an ethical manner. An academic colleague agreed this was a good idea ‘just in case’ it was questioned by others, in particular by a journal editor when seeking to publish (which indeed it was). However, we view this as unnecessary given the activity was not classed as research and therefore not in the remit of the IRB. The IRB provided written agreement that ethical review was not required for this project and the project team agreed a standard engagement risk assessment would consider and address any ethical issues.

### Example 2

*Co-producing an educational online resource for school children – request from publication to seek ethical approval for the project*.

This co-production project working with researchers, a PI and PE professional, school teachers and web designers aimed to develop an educational online resource for school age children and their teachers. This interdisciplinary team of experts were involved in four online workshops to support the delivery and development of a website that would support teachers and enhance learning. All individuals involved fully signed up to the coproduction focus of the project and provided verbal agreement to take part in the workshops and off-line discussions. However, when trying to publish the co-production process, the journal editor stressed that according to journal policy ‘research involving human subjects, human material or human data must have been approved by an appropriate ethics committee’.

The authors explained that the project did not involve human subjects, human material or human data (as it was not research) and therefore in their opinion did not require ethical approval. The journal editor disagreed, arguing that the project was a research study that collected and analysed data, and that the teachers and web designers involved in this project were human participants of the study and data had been generated of their opinions. The editor recommended seeking either retrospective ethical approval or else removing all human data. The team saw no alternative but to withdraw their original manuscript and submit the work elsewhere.

### Example 3

*Co-production project involving people from minority ethnic backgrounds in discussion about inclusive health research – project investigators not comfortable including quotes from public contributors due to lack of informed consent.*

This project involving researchers, an artist, charity project workers serving the most ethnically diverse ward in Wales and local residents aimed to answer the question: ‘How can people from minority ethnic backgrounds influence health research in terms of both what and how this research is done?’ Eight co-production workshops drawing on the participatory democracy approach were held and delivered a set of recommendations for the health research community. In advance of these workshops, a university IRB Chair helped to clarify that ethical approval was not needed.

When publishing this work, researchers did not include quotes obtained from the workshops as informed consent had not been sought (as it was not research) [23]. On reflection, the authors would like to have gained agreement for the residents’ quotes to be used, in the absence of the requirement for documented informed consent.

## Identified exceptions

Whilst PI and PE activities do not generally require ethical approval, there are at least two example scenarios where approval *is* required. Firstly, for example, when systematically comparing two methods of involvement and/or engagement to understand which is better i.e. answering a research question about PI/PE to produce generalisable or transferable findings. Secondly, when public members come into direct contact with study participants or their data e.g. if assisting with conducting research interviews or analysing the transcripts. In this situation, ethical approval is required because human participants are involved in the research.

## Recommendations for moving forwards

We encourage the research community, including researchers, publishers, reviewers, funders and ethics committees to better appreciate the difference between PI and PE and research involving human participants; to recognise that all involved stakeholders operate within professional boundaries; and to work together to agree an alternative accepted approach when the PI and PE activity raises ethical considerations (e.g. when working with vulnerable groups or publishing of public contributor quotes). The responsibility of determining whether research ethic approval is required falls on the individuals/team planning the activity. We understand that it is tempting to seek research ethical approval for PI and PE activity ‘just in case’ or ‘to be on the safe side’, but we do believe this is detrimental for several reasons including:

- Sustains the confusion between qualitative research and PI and PE activity, and the different purposes of each.
- Wastes valuable researcher and committee time and resources.
- Undermines the importance of the research ethics approval process.
- Delays PI and PE activities in the research process, potentially leading to missing out on the benefits of earlier involvement.
- Undermines coproduction principles such as equality and shared responsibility between researchers and members of the public. The process of acquiring ethical approval itself asserts a hierarchy whereby a researcher is identified as Chief/Principal investigator, and other members of the team are listed below an identified leader.
- Acts as an additional barrier and disincentive to researchers carrying out PI and PE activity.

**Fig. 1:**
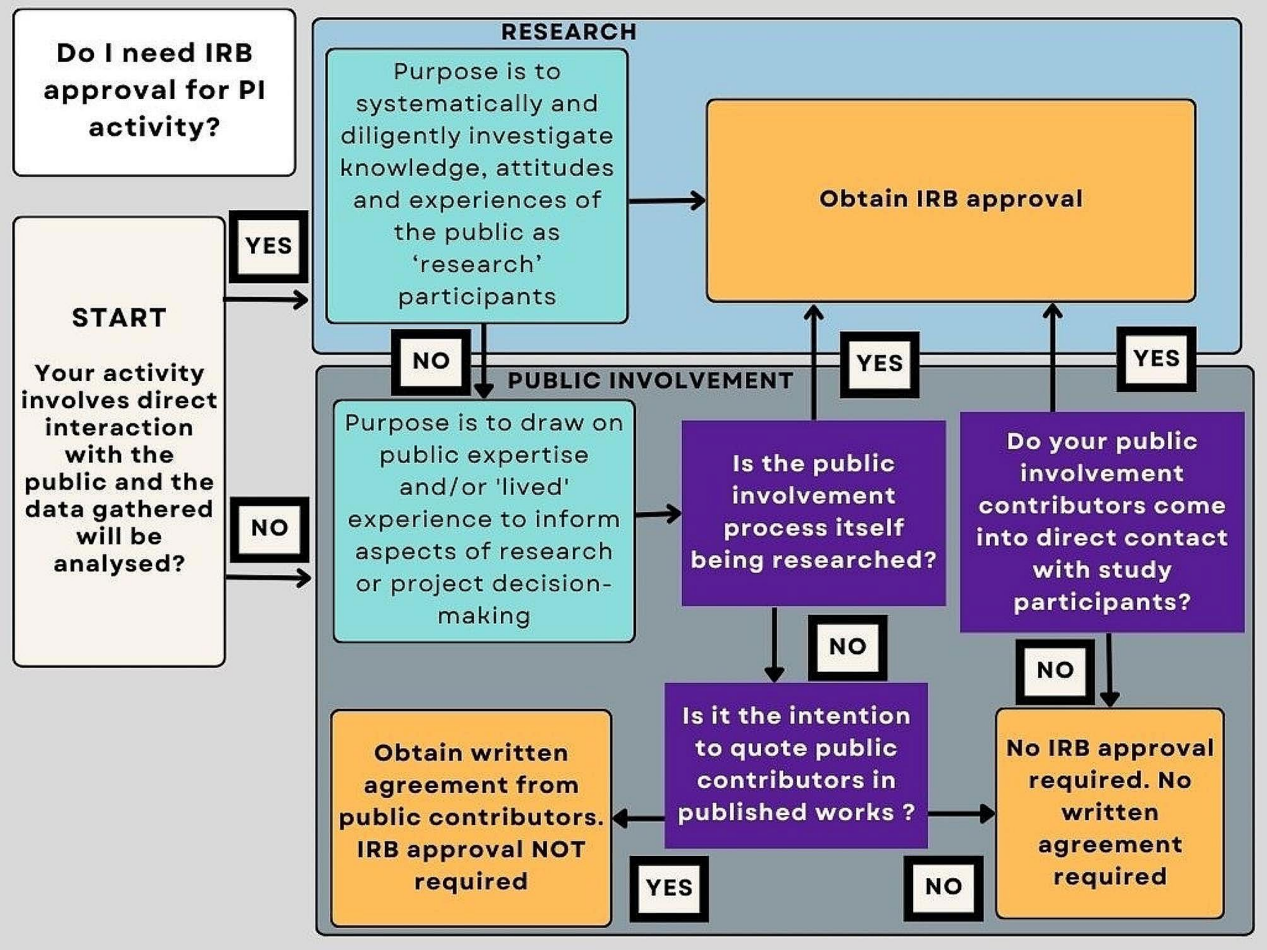
Simple flow diagram to support researchers to decide on the need for research ethical approval via an IRB

There is a need to address this growing problem, via education and generating solutions acceptable to the community as a whole, providing confidence in decisions made and assurances that the health and safety and any risks associated with the proposed PI and PE activity have been carefully considered and approved. Here we present key recommendations for those conducting public involvement and engagement activities based on our internal guidance (Appendix 1) for alternative courses of action moving forwards when faced with these challenges.

1. **Purpose** - Consider the purpose of the activity. Is it to answer a scientific or clinical question (research) or help shape, guide or disseminate the research (PI/PE)? If you are unsure if your project is research, you can consult the UK Health Research Authority’s ‘Is my study research’ decision tool. Following response to three questions, (1. Are the participants in your study randomised to different groups? 2. Does your study protocol demand changing treatment/care/services from accepted standards for any of the patients/service users involved? 3. Is your study designed to produce generalisable or transferable findings? ) The tool confirms if your study would be considered as research. This result can be downloaded and further advice can be sought [24]. The HRA table ‘Defining Research’ can also help provide clarification [25].
2. Internally, a simple flow diagram (Fig. 1) has been created to support researchers in making a decision on the need for research ethics approval when carrying out public involvement activity.

3) **Risk assessment** – To ensure PI and PE activities are conducted in a safe and ethical manner, particularly when engaging and/or involving ‘vulnerable’ groups, refer to published guidance on conducting ethical PI&E [12–16], consider completing a specifically designed PI and PE risk assessment (See Appendix 2 for an example) or using the PIRIT tool [26]to assess your planned activities and undertake adequate training (See Appendix 2 for an example). Use the same considerations as you might for research or teaching e.g. what to do if an individual becomes upset in a discussion group, how to support them, where to refer them. Also consider safety, protection of anonymity and confidentiality of personal data. Use the UK Standards on Public Involvement [4] to guide your thinking around accessibility and inclusivity when completing the assessment. If possible, involve a public contributor and have this signed off by a senior academic/responsible member of staff in your organisation.
4) **Adequate information and agreement to take part** – Ensure that public members being invited to take part in PI and PE activity agree for you to use their anonymous quotes in any output. But understand that standard Participant Information Sheets and Informed Consent Forms are not required as formal consent is not required.
5) **Language** – To avoid confusion for reviewers and publishers, think carefully about the language you use to describe your PI and PE activities. For example, use the term ‘discussion group’ rather than ‘focus group’; refer to members of the public as ‘attendees’ not ‘participants’ and input as ‘contributions’ rather than ‘data’; and ‘summarising key points or themes’ as opposed to ‘thematic analysis’ when describing your activities (if that is indeed what you are doing).
6) **Written confirmation** – Some institutions have established infrastructure to support researchers through a self-assessment process for governance and ethics, providing a confirmatory statement as to whether ethical approval is required if challenged by funders and publishers [27]. However, not all institutions have this facility and until this area of contention is resolved, some individuals may wish to seek written confirmation from their local IRB. In our experience, a letter confirming approval is not required is acceptable by journal editors. Liaise with your local IRB to determine if this is within their remit.
7) **Training** – The development and inclusion of training for researchers and support staff is required on when to seek ethical approval and how to effectively manage ethical, risks, and health and safety aspects of PI and PE in a considered, widely accepted and non-burdensome way.

## Conclusions

Our experience suggests that ambiguity remains in the academic community about whether ethical approval is needed for PI and PE activities. We believe this stems from (1) the grey area between qualitative research and PI and PE activities; (2) seeking approval ‘just in case’ they are requested by funders, publishers or authorities (based on previous experience) (3) funders, publishers and authorities not being clear in the distinction and equally asking for approval ‘just in case’ and (4) a lack of an alternative recognised way to evidence that ethical issues have been considered and mitigated against. We have used real world examples to demonstrate the issues encountered in a single institution and make several recommendations aimed at researchers for addressing this area of contention going forward. We appreciate that our views may be framed by our experience of conducting PI&E in a healthcare context and in the UK, and the experiences of researchers in other disciplines and countries may vary significantly.

We hope this commentary triggers debate in the community to highlight, educate and clarify the position surrounding research ethics and PI and PE activity amongst researchers, funders and journal editors. Our experience shows that this issue is effectively acting as a barrier to researchers conducting PI and PE activity and publishing PI and PE learning. An alternative recognised process needs to be established by the community to resolve this growing detrimental development.

### Electronic supplementary material

Below is the link to the electronic supplementary material.

Supplementary Material 1

Supplementary Material 2

## Data Availability

Not applicable.

## Declarations

### Authors information

CN is Academic Lead for Public Involvement and Engagement in the Centre for Trials Research, Cardiff University. SH is the Public Involvement and Engagement Manager for the School of Medicine, Cardiff University, alongside researchers ME and NJW who are the Joint Academic Leads for Public Involvement and Engagement in the School of Medicine, Cardiff University. ME is also the Engagement Lead for the Systems Immunity Research Institute at Cardiff University and the Engagement Secretary for the British Society for Immunology. JF was the Lead Public Contributor in the School of Medicine at the time of writing.

## Abbreviations

HRA: Health Research Authority
IRB: Institutional review board
PE: Public engagement
PI: Public involvement
REC: Research Ethics Committee
REF: Research Excellence Framework
