# Supplementary material for: Public involvement and engagement in scientific research and higher education: the only way is ethics?
Source: Res Involv Engagem. 2024 May 31;10:50. doi: 10.1186/s40900-024-00587-x (PMC11140937; doi:10.1186/s40900-024-00587-x)
Supplement: Supplementary file 1 — Supplementary Material 1 [file 40900_2024_587_MOESM1_ESM.docx]

**Risk assessment**

**Risk Assessment: Public Involvement Activity**

| **Name of School/Department:** |  | **Signature of assessor:** |  |
| --- | --- | --- | --- |
| **Location to be visited:** |  | **Date:** |  |
| **Duration of public involvement activity (include start/end dates):** |  | **Signature of manager/academic lead:** |  |
| **Contact numbers (Emergency and local):** |  | **Date:** |  |
| **University emergency contact:** | Security hotline on **029 2087 4444** for 24 hour emergency assistance helpline if you need assistance. | | |
| **Brief summary/purpose of public involvement activity:** |  | | |
| **Signature(s) of those involved in delivery of involvement activity:** |  | | |

**People: public contributors, service users, volunteers, staff, the public, others**

| **What are the hazards?** | **Who might be harmed and how?** | **What are you doing already to support and/or reduce the risk?** | **What further action is necessary to reduce the risk?**  **Consider: Support to be offered; Reduce/remove the risk; Minimize the harm; share the liability** | **Action by whom?** | **Action by when?** | **Date completed** |
| --- | --- | --- | --- | --- | --- | --- |
| ***Example:***  *Discussion group involves sensitive discussion matter* | *Public contributors may get upset talking about lived experience of condition* | *Carefully draft discussion group questions to ensure they do not have a personal focus and are as neutral as possible.* | *Minimise harm: Facilitator to acknowledge the person is showing signs of being upset. Offer that they take a break but also make them feel comfortable to stay. Acknowledge that is a difficult topic to discuss and encourage that they take their time.* | *Discussion group facilitator* | *As needed on day of discussion group* | *05/06/2023* |
| ***Example:***  *Consider any potential safeguarding issues if the activity includes children/young people or vulnerable adults.* | *Recognise the potential for a public contributor to disclose a safeguarding issue* | *Ensure you have enough support to be able to deal with any disclosure and avoid disruption of the main aims of the public involvement activity. Potentially book a separate room nearby that could be used should this situation arise.* | *Be familiar with Cardiff University’s Safeguarding Policy and be aware of who your safeguarding officers are.* | *PPI lead* | *In advance of the PPI taking place* |  |
|  |  |  |  |  |  |  |
|  |  |  |  |  |  |  |
|  |  |  |  |  |  |  |
|  |  |  |  |  |  |  |
|  |  |  |  |  |  |  |
|  |  |  |  |  |  |  |

**Property: premises, equipment, vehicles, infrastructure, etc.**

| **What are the hazards?** | **Who might be harmed and how?** | **What are you doing already to reduce the risk?** | **Do you need to do anything else to manage this risk?**  **Consider: Reduce/remove the risk; Minimize the harm; share the liability** | **Action by whom?** | **Action by when?** | **Date completed?** |
| --- | --- | --- | --- | --- | --- | --- |
| ***Example:*** *Facilitator’s technology breaks* | *Attendees could be frustrated and leave the discussion group* | *Facilitator has both a PC and laptop available and will set them up to quickly switch between the two. Mobile phone also available in case of Wi-Fi outage.* | *No* |  | *15/9/2023* | *3/9/2023* |
|  |  |  |  |  |  |  |
|  |  |  |  |  |  |  |
|  |  |  |  |  |  |  |
|  |  |  |  |  |  |  |
|  |  |  |  |  |  |  |

**Goodwill: reputation with users, supporters, funders, etc.**

| **What are the hazards?** | **Who might be harmed and how?** | **What are you already doing?** | **Do you need to do anything else to manage this risk?**  **Consider: Reduce/remove the risk; Minimize the harm; share the liability** | **Action by whom?** | **Action by when?** | **Date completed?** |
| --- | --- | --- | --- | --- | --- | --- |
| ***Example:*** *Facilitator insensitivity* | *The attendees could be offended by lack of sensitivity around difficult topic* | *Facilitator to have appropriate training in subject topic. As well as experience in interviewing service users and staff about the subject topic and in facilitating engagement discussion groups.* |  | *Facilitator* | *Prior to first PPI group session* |  |
|  |  |  |  |  |  |  |
|  |  |  |  |  |  |  |
|  |  |  |  |  |  |  |

**Adverse events: hazardous events and substances**

| **What are the hazards?** | **Who might be harmed and how?** | **What are you already doing?** | **Do you need to do anything else to manage this risk?**  **Consider: Reduce/remove the risk; Minimize the harm; share the liability** | **Action by whom?** | **Action by when?** | **Date completed?** |
| --- | --- | --- | --- | --- | --- | --- |
| ***Example***  *Visitors viewing jars with ethanol-fixed parasites* | *Ethanol fumes, accidently broken glassware* | *Visitors will need to be supervised and not allowed to handle glassware with Ethanol* | *University staff to be familiar with evacuation arrangements* | *Project leader, all staff and volunteers* | *15/9/2023* | *3/9/2023* |
|  |  |  |  |  |  |  |
|  |  |  |  |  |  |  |
|  |  |  |  |  |  |  |

**Health and fitness, personal safety**

| **What are the hazards?** | **Who might be harmed and how?** | **What are you already doing?** | **Do you need to do anything else to manage this risk?**  **Consider: Reduce/remove the risk; Minimize the harm; share the liability** | **Action by whom?** | **Action by when?** | **Date completed** |
| --- | --- | --- | --- | --- | --- | --- |
| ***Example:***  *Contaminated drinking water/poor food hygiene* | *Staff, volunteers could catch food poisoning from poor food hygiene or contaminated drinking water* | *Staff and volunteers are briefed on food hygiene and are advised to only drink bottled water; anti-bacterial hand gel used frequently* | *Project leader reminds volunteers to use anti-bacterial gel and be diligent about cleanliness* | *All, monitoring by supervisor* | *15/9/2023* | *3/9/2023* |
|  |  |  |  |  |  |  |
|  |  |  |  |  |  |  |
|  |  |  |  |  |  |  |
|  |  |  |  |  |  |  |
|  |  |  |  |  |  |  |
|  |  |  |  |  |  |  |
|  |  |  |  |  |  |  |

**Other: please include any other identified risks that do not fall into the categories above**

| **What are the hazards?** | **Who might be harmed and how?** | **What are you already doing?** | **Do you need to do anything else to manage this risk?**  **Consider: Reduce/remove the risk; Minimize the harm; share the liability** | **Action by whom?** | **Action by when?** | **Date completed** |
| --- | --- | --- | --- | --- | --- | --- |
| ***Examples:*** *Natural disasters* |  |  |  |  |  |  |
|  |  |  |  |  |  |  |
|  |  |  |  |  |  |  |
|  |  |  |  |  |  |  |
|  |  |  |  |  |  |  |

**Examples of risk by category**

| **People** | **Property** | **Goodwill** | **Hazardous events** | **Health and Fitness, personal safety** | **Other** |
| --- | --- | --- | --- | --- | --- |
| Disclosures | Fire | Cultural sensitivity | Hazardous activities e.g extreme sports | Crime | Terrorism |
| Child protection issues | Contaminated food | Working with people who have protected characteristics | Exposure to hazardous substances/chemicals | Violence and Aggression | Natural disasters |
| Slips, trips and falls | Contaminated water | Legal differences | Operation of machinery | Medical Emergencies | Political unrest |
| Lost persons | Utilities | Religious differences |  | Lone working |  |
| DBS checks | Equipment | Appropriate clothing |  | Taking photographs |  |
| Community work with known high risk groups of clients (drug abusers, homeless) | Slippery floors | Politically sensitive issues |  | Night travel/night working |  |
|  | Trailing cables |  |  | Visiting homes |  |

Advice and guidance on completing this form is available from the School of Medicine Public Involvement and Engagement team, please contact [medicengagement@cardiff.ac.uk](mailto:medicengagement@cardiff.ac.uk)

Please note: When completing the template please only include acceptable risks. For more information on completing a risk assessment please contact the Safety and Staff Wellbeing team: [Safety@cardiff.ac.uk](mailto:safety@cardiff.ac.uk?subject=contact)
