# Supplementary material for: Public involvement and engagement in scientific research and higher education: the only way is ethics?
Source: Res Involv Engagem. 2024 May 31;10:50. doi: 10.1186/s40900-024-00587-x (PMC11140937; doi:10.1186/s40900-024-00587-x)
Supplement: Supplementary file 2 — Supplementary Material 2 [file 40900_2024_587_MOESM2_ESM.docx]

**
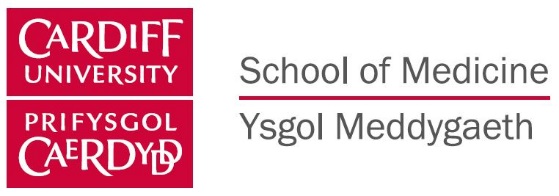
**

**Do I need research ethics approval for Public Involvement activities? In short, No.**

The following guidance has been drafted in response to growing confusion around whether public involvement activities require ethical approval, particularly when academics wish to publish details and outcomes of their public involvement activity. Currently, researchers are commonly seeking ethics approval, ‘just in case’ or ‘to make sure we can publish;’ this is both an unnecessary process and reflects a poor understanding of the nature of public involvement in the research community.

| **Research (CU definition)**  Research means any project which attempts to  derive generalisable new knowledge  or apply existing knowledge, including studies that aim to generate hypotheses, as well as studies that aim to test them. | **Public Involvement (NHS HRA definition)**  Public involvement in research is research carried out ‘with’ or ‘by’ members of the public rather than ‘to’, ‘about’ or ‘for’ them. It means that patients or other people with relevant experience contribute to how research is designed, conducted and disseminated. It does not refer to research participants taking part in a study. |
| --- | --- |

Public involvement in research is an opportunity for members of the public to share their ‘lived’ experiences of health conditions or other circumstances to inform the priorities, design, delivery and implementation of research and education, so that it is more relevant to people’s needs.

Public involvement is important for undertaking safe, relevant and ethical research and should be a routine and normal part of the research process. In the School of Medicine, public involvement in both research and teaching are recognised aspects of academic work that play a vital role in enhancing the School’s core activities. It is important to acknowledge that public involvement activities in research are not a research method but a process for involving patients as partners in the planning and conduct of research and education. Public involvement activities therefore do not need Research Ethics Committee (REC) review.

The UK Health Research Authority’s (HRA) policy framework for health and social care research reinforces this position, stating that ‘the involvement of patients, service users or the public in the design, management or conduct of research… is ***not*** *subject to approval* (e.g. from a research ethics committee).’ (1)

The National Research Ethics Service (NRES) and INVOLVE have also written a statement to clarify the position of ethics and PPI. Their advice is as follows: “The active involvement of patients or members of the public does not generally raise any ethical concerns for the people who are actively involved, even when those people are recruited for this role via the NHS. This is because they are not acting in the same way as research participants. They are acting as specialist advisers, providing valuable knowledge and expertise based on their experience of a health condition or public health concern.” (2)

**Grey Areas**

However, there are many ‘grey areas’ and guidance from recent publications can help to clarify research vs public involvement activities. For example:

One of the key areas of confusion is qualitative research vs public involvement activities. Guidance is provided by Hanley et al (2019) in ‘Qualitative research and patient and public involvement in health and social care research: What are the key differences?’ (learningforinvolvement.org.uk). In particular, consultation with a number of people at one time, usually referred to as ‘focus groups’, can cause particular problems (3). A Canadian health organisation (Maritime SPOR Support Unit: <https://mssu.ca/>) recommends using two different terms:

Focus groups refer to research activities, and discussion groups refer to public involvement activities. We agree with this suggestion and propose that in focus groups, the people taking part will be research participants. Their contributions will usually be transcribed verbatium and treated as data, to be systematically analysed. Focus groups require REC approval and the participant to receive a Participant Information Sheet and give informed consent. On the contrary, in a discussion group, the people present will be ‘involved' in shaping the research (not participants of the research). Their contributions will usually be noted as key points and not treated as data or systematically analysed. They will be used to guide the research. Discussion groups do not need REC approval. Attendees should be given some information about the activity beforehand and may be asked to give consent, but not in the structured way as for focus groups. There is some debate about whether direct quotes can be used in a publication from people in a discussion group, and we advise gaining documented consent if you wish to do this e.g. an email from the group member consenting to anonymous quotes being used in a publication.

In some cases, researchers will need to combine public involvement activities with a qualitative research approach. For example, you may wish to interview new mothers as research participants to get their views on motherhood. This would require REC approval. But prior to interviews, you may want to involve new mothers in a discussion group to help shape the topic guide for the interviews. This would not need REC approval. See [Morgan et al](https://doi.org/10.1186/%20s40900-016-0023-1) (4).

It is now a requirement of many funders to involve patients and other members of the public in shaping and contributing to grant applications. As with post-grant activities, these pre-grant public involvement activities do not require REC approval.

Utilising research techniques, for example, Delphi surveys in a public involvement activity does not mean that REC approval is required. However, this does depend on the REC you are reporting to, as we are aware of external examples when it has been required by a REC.

One instance where REC approval might be required, is if you are researching the public involvement process itself. For example, you may wish to compare the effectiveness of conducting individual vs group involvement activities – this could be considered research and may need ethics approval – consider the definition of research in the introduction to this guidance. Also, when public involvement contributors come into direct contact with study participants, for example, public contributors are actively involved as researchers and conduct interviews with study participants. The REC will need to consider the involvement as an element of the ethical consideration and approval.

**Ethical public involvement**

Whilst public involvement activities do not need REC approval, they **DO** still need to be conducted in a considered and ethical manner and it is likely you will need to make many of the same provisions and adjustments for those involved as you would for participants.

**Publishing/funding public involvement activities**

It is becoming common to publish involvement activities, particularly those that are novel or are with under-served populations etc and the School encourages this. Unfortunately, in our experience, some journal editors do not fully understand public involvement and ask for REC approval. In this instance, the School Research Ethics Committee are happy to provide confirmation that a project did not require ethical review via a formal letter.

Some public involvement funders have also required evidence that all appropriate support has been taken into consideration when involving members of the public in an activity (especially if they’re vulnerable). Here, the following recommendations are advised:

- Complete a public involvement risk assessment (here) for your activity and send with your project proposal to your line manager for sign off.
- Provide signed assessment to funder.
- If funder is not satisfied with this evidence, please contact [medicengagement@cardiff.ac.uk](mailto:medicengagement@cardiff.ac.uk) for assistance.


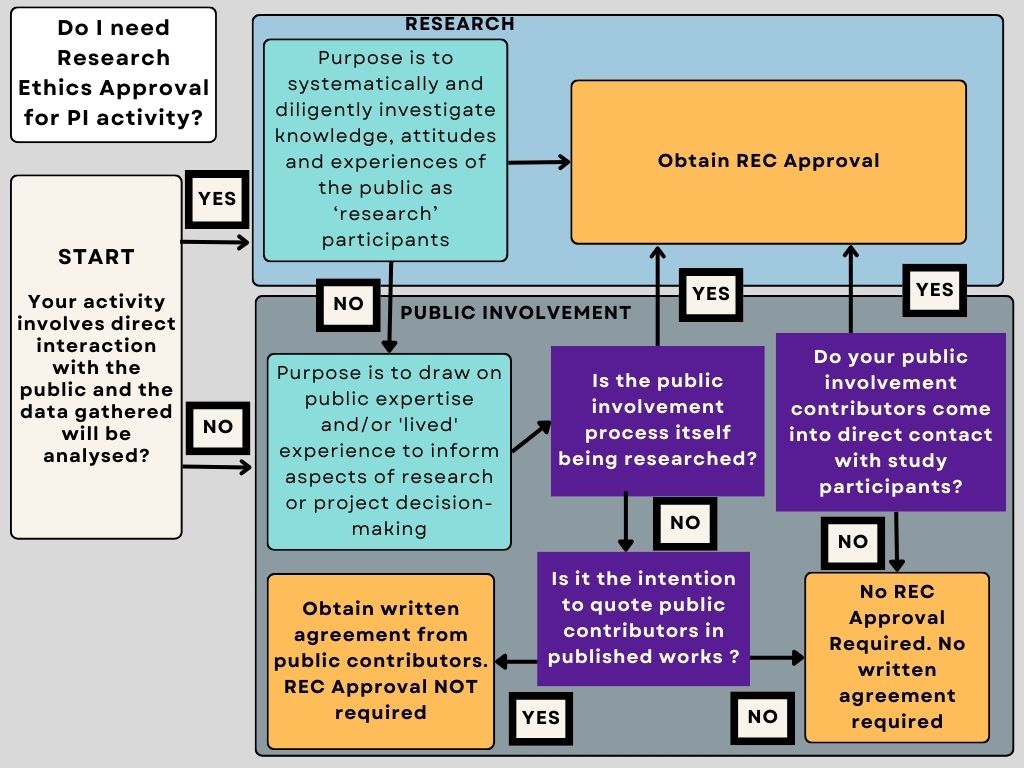


- If after reading this guidance you are still unclear, please review the following REC guidance: <https://intranet.cardiff.ac.uk/staff/supporting-your-work/research-support/research-integrity-and-governance/research-ethics/ethical-review/school-research-ethics-committees-srecs/school-of-medicine>

This guidance has been drafted by Sarah Hatch and Dr Claire Nollett, reviewed by Health and Care Research Wales and endorsed by the School of Medicine Research Ethics Committee.

**References**

1. Do I need NHS REC approval? This tool helps you to work out whether your study needs approval from an NHS REC. [Do I need NHS Ethics approval? (hra-decisiontools.org.uk)](https://www.hra-decisiontools.org.uk/ethics/)
2. [RDS_PPI-Handbook_2014-v8-FINAL-11.pdf (nihr.ac.uk)](https://www.rds-yh.nihr.ac.uk/wp-content/uploads/2015/01/RDS_PPI-Handbook_2014-v8-FINAL-11.pdf) (Please refer to page 9).
3. Doria et al. Research Involvement and Engagement (2018) 4:19 <https://doi.org/10.1186/s40900-018-0102-6>
4. Morgan, H, Thomson, G, Crossland, N, Dykes, F, Hoddinott, P, on behalf of the ‘BIBS’ study team, 2016, Combining PPI with qualitative research to engage ‘harder-to-reach’ populations: service user groups as co-applicants on a platform study for a trial, *Research Involvement and Engagement*, 2, 7, [https://doi.org/10.1186/ s40900-016-0023-1](https://doi.org/10.1186/%20s40900-016-0023-1)
